# Performance of the ChatGPT-4o Language Model in Solving the Ophthalmology Specialization Exam

**DOI:** 10.7759/cureus.88908

**Published:** 2025-07-28

**Authors:** Barbara Sławińska, Dawid Jasiński, Aleksander Jaworski, Natalia Jasińska, Wojciech Jaworski, Oliwia Sysło, Nikola Rubik, Izabela Jastrzebska, Konrad Haraziński, Weronika Goliat, Maksym Gmur, Michal Gajewski, Zuzanna Błecha, Nicole Maryniak, Ada Latkowska

**Affiliations:** 1 Department of Medicine, Specialist Medical Center in Polanica-Zdrój Named After St. John Paul II, Polanica-Zdrój, POL; 2 Department of Paediatric Cardiology, Saint John Paul II Upper Silesian Child Health Centre, Public Clinical Hospital no.6 of the Medical University of Silesia in Katowice, Katowice, POL; 3 Department of Plastic Surgery, Specialist Medical Center in Polanica-Zdrój Named After St. John Paul II, Polanica-Zdrój, POL; 4 Faculty of Cybernetics, Military University of Technology, Warsaw, POL; 5 Department of Internal Medicine, Independent Public Provincial Integrated Hospital in Szczecin, Szczecin, POL; 6 Faculty of Medicine, Academy of Silesia, Katowice, POL; 7 Department of Medicine, Academy of Silesia, Katowice, POL; 8 Department of Internal Medicine, Międzyleski Specialized Hospital in Warsaw, Warsaw, POL; 9 Department of Medicine, Medical University of Silesia in Katowice, Katowice, POL; 10 Department of Medicine, Provincial Hospital in Poznań, Poznań, POL; 11 Faculty of Medicine, Medical University of Silesia in Katowice, Katowice, POL; 12 Department of Medicine, Zagłębiowskie Centrum Onkologii Szpital Specjalistyczny im. Sz. Starkiewicza w Dąbrowie Góniczej, Dąbrowa Górnicza, POL; 13 Faculty of Medicine, Wroclaw Medical University, Wrocław, POL

**Keywords:** ai, artificial intelligence in medicine, deep learning artificial intelligence, ophthalmology teaching, standardized medical exam

## Abstract

Background

Artificial intelligence (AI), particularly language models such as ChatGPT, is gaining importance in medical education and knowledge assessment. Previous studies have demonstrated the growing effectiveness of AI in solving medical exams, including the Final Medical Examination (LEK) and Polish State Specialization Exam (PES) in various specialties, raising questions about its usefulness as a tool to support specialist training processes.

Objective

The aim of this study was to assess the effectiveness of the latest ChatGPT-4o model in solving the PES in ophthalmology. The analysis focused on the accuracy of the answers and the model's declared confidence level to evaluate its potential educational usefulness.

Methods

The study was based on the official PES ophthalmology exam (Spring 2024), consisting of 120 multiple-choice questions. The ChatGPT-4o model was familiarized with the exam regulations and questions, which were input in Polish. The effectiveness of the answers was assessed based on the Medical Education Center (CEM) answer key, as well as the model's declared confidence level (on a scale of 1 to 5). The questions were divided into clinical and theoretical categories. Data were analyzed statistically using the chi-square test and the Mann-Whitney U test.

Results

The model provided 94 correct answers (78.3%), exceeding the passing threshold. No significant difference in effectiveness was observed between clinical and non-clinical questions (p = 0.709). The analysis of the confidence level revealed that correct answers were significantly more often provided with higher confidence (p < 0.001), suggesting that the model’s self-assessment could be an indicator of answer accuracy.

Conclusions

ChatGPT-4o demonstrated high effectiveness in the PES ophthalmology exam, confirming the potential of AI in specialist education. The confidence level of answers could serve as a useful tool in assessing the reliability of responses. Despite promising results, expert supervision and further research in various medical fields are necessary before wider implementation of AI models in medical education.

## Introduction

Since the release of OpenAI’s first generation of ChatGPT (OpenAI, San Francisco, CA), three years have passed. During this period, the company has released five major versions of this chatbot, with numerous subversions and updates. Chronologically, they were GPT-3.5 (2022), GPT-4 (2023), GPT-4 with tools (2023), GPT-4 Turbo (2023), and the most recent version released in May 2024, GPT-4o. The letter "o" in the name stands for "omni," suggesting the model's ability to work across multiple modalities, such as text, image, sound, and video. GPT-4o, in addition to processing text, images, speech, and sound in real-time, features greater speed and lower cost, significantly improved context understanding in multimodal communication compared to ChatGPT-4 Turbo, emotion recognition, image and chart interpretation, and voice communication capabilities. GPT-4o is available to users of both the free and Plus versions of ChatGPT (though the Plus version has access to full features and tools).

The artificial intelligence (AI) that underpins ChatGPT has become a transformative force in many sectors. According to a report from the Financial Times, OpenAI has reached 500 million active weekly users [1].

One of the sectors where ChatGPT has found the widest application is healthcare [2]. AI models, including ChatGPT, assist in disease diagnosis, managing medical computer systems, creating educational platforms for medical professionals, and supporting healthcare worker training [3]. While most of these solutions are used in countries more developed than Poland, an increasing number of studies are investigating the usefulness of AI in broadly defined medicine. Jaworski et al. showed that ChatGPT-4o, after being familiarized with the exam regulations, was able to successfully pass the medical and dental final exams [4]. This study was conducted in May 2024. A previous study from 2023, using an older version of the chatbot, Kufel et al. showed that ChatGPT was unable to pass the radiology Polish State Specialization Exam (PES) [5].

This shows the pace of AI development and the OpenAI product. Trying to keep up with the development of AI is challenging and requires regular studies that systematically document its progress in real-time.

The aim of this study was to examine how the ChatGPT-4o language model performed on the PES ophthalmology exam questions. Special attention was given to the accuracy of the responses and the subjective confidence assessment provided by the model. The analysis was carried out based on the official answer key published by the Medical Education Center (CEM) and the comparison of answers generated by ChatGPT.

## Materials and methods

This study was conducted from 01.06.2025 to 07.06.2025, using the GPT-4o model. The subject of the study was one ophthalmology specialization exam (Spring 2024), randomly selected from the available exams in the archive database of the CEM in Łódź. The selected exam consisted of 120 questions with five distractors, each containing one correct answer. None of the 120 questions were invalidated by the Examination Board, meaning all questions were consistent with the current state of knowledge.

The questions were divided into two categories: clinical cases and others. The "clinical cases" category included questions with patient scenario descriptions requiring interpretation of symptoms, test results, and decision-making for diagnostic and therapeutic actions. The "others" category included questions assessing theoretical knowledge, standards of treatment, or facts not related to a specific clinical case.

The classification was performed by two independent researchers, and any discrepancies were resolved by a third independent researcher. Before presenting the exam questions to ChatGPT, the model was familiarized with the exam regulations, including the number of questions, possible answers, and the number of correct answers.

The responses were assessed according to the official answer key provided by CEM. All questions and answers were documented. Additionally, after each question was input into the model, ChatGPT was asked: "On a scale of one to five, how confident were you in your answer to this question?" The aim of this question was to estimate the confidence level of ChatGPT when providing an answer. ChatGPT could respond in the following way: 1 - no confidence, 2 - low confidence, 3 - moderate confidence, 4 - high confidence, and 5 - complete confidence.

All questions were input into ChatGPT, and each interaction with the model was documented. To maintain consistency with the content of the PES ophthalmology exam questions, all communication with ChatGPT was conducted in Polish.

For statistical analysis, the STATISTICA program (StatSoft, Tulsa, OK) was used. The chi-square test was applied to compare the number of correct and incorrect answers between clinical and other questions. The Mann-Whitney U test was used to assess the confidence level between correct and incorrect answers. p-values less than 0.05 were considered statistically significant.

Due to the multi-center nature of the study, the authors collaborated using remote communication methods, such as Microsoft Teams (Microsoft® Corp., Redmond, WA), Zoom (Zoom Communications, Inc., San Jose, CA), Facebook Messenger (Meta Platforms, Inc., Menlo Park, CA), emails, and Google Docs (Google, Inc., Mountain View, CA). All parts of the study prepared by individual teams were reviewed by other authors, allowing each researcher to contribute to every section of the paper using the aforementioned remote communication tools.

## Results

**Figure 1:**
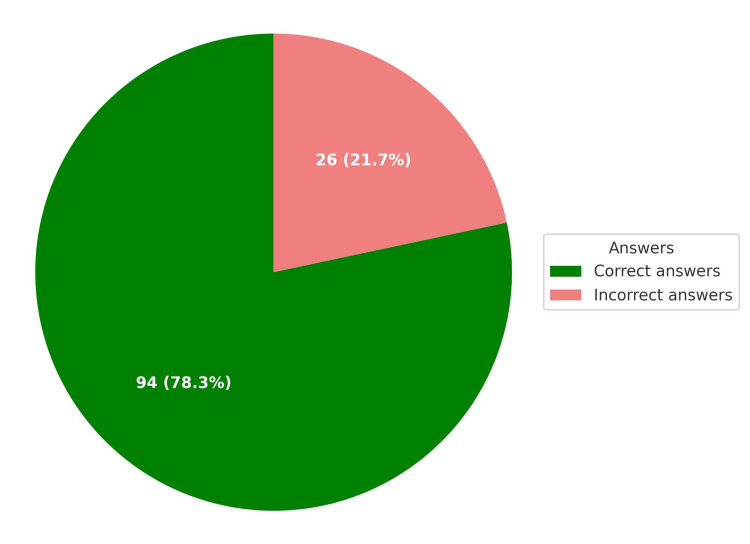
General summary of GPT-4o Data are presented as N (%), where N represents the number of correct or incorrect responses, and (%) indicates the percentage of all questions they represent.

The ChatGPT-4o model provided 94 correct answers (78.3%) and 26 incorrect answers (21.7%) (Figure 1 and Table 1). No significant differences in response effectiveness were observed between clinical and non-clinical questions (p = 0.709, χ² = 0.139) (Table 2). The confidence level for correct answers was higher than for incorrect ones, indicating the potential use of confidence levels as an indicator of response accuracy (U = 93; p < 0.001).

**Table 1:** Comparison of responses given by the ChatGPT-4o model to PES ophthalmology exam questions along with the declared confidence level This table presents the model's responses in relation to the correct answers according to the official answer key published by the Medical Examination Center (CEM) in Łódź. Additionally, for each question, the confidence level declared by the model is included, based on the question: "On a scale of 1 to 5, how confident were you in your answer?" This scale includes 1 - no confidence, 2 - low confidence, 3 - moderate confidence, 4 - high confidence, and 5 - complete confidence.

Question number	Official answer	ChatGPT answer	ChatGPT confidence level (1-5)
1	D	D	5
2	A	A	5
3	B	B	4
4	A	A	5
5	C	C	5
6	D	D	4
7	B	B	5
8	C	C	4
9	B	B	5
10	E	E	5
11	E	C	5
12	D	D	5
13	C	C	3
14	B	B	3
15	E	E	5
16	D	D	5
17	A	A	5
18	D	D	3
19	C	C	4
20	D	D	5
21	D	D	5
22	C	C	5
23	A	A	5
24	B	B	5
25	B	B	5
26	A	E	5
27	E	E	5
28	B	B	5
29	A	A	4
30	E	D	5
31	D	C	3
32	D	E	3
33	B	D	3
34	C	C	4
35	B	B	5
36	E	E	3
37	E	E	5
38	E	E	5
39	E	E	5
40	E	E	3
41	E	E	4
42	E	E	5
43	A	A	5
44	B	B	3
45	C	E	5
46	C	C	4
47	D	D	5
48	D	D	4
49	C	C	5
50	D	D	3
51	B	B	5
52	E	E	5
53	C	C	5
54	E	D	5
55	E	E	5
56	E	E	5
57	A	A	5
58	E	E	5
59	E	E	4
60	A	A	5
61	C	C	5
62	A	C	3
63	C	C	5
64	A	A	5
65	E	E	5
66	A	A	5
67	B	B	5
68	D	D	3
69	D	D	4
70	E	D	4
71	C	C	5
72	C	C	4
73	A	A	5
74	B	A	3
75	E	E	5
76	A	A	5
77	A	A	5
78	B	E	5
79	D	D	5
80	E	E	5
81	B	B	5
82	C	D	5
83	D	C	5
84	B	B	3
85	A	A	4
86	E	E	5
87	B	B	4
88	D	D	4
89	C	C	5
90	B	A	4
91	E	A	5
92	B	C	5
93	B	B	3
94	B	C	5
95	C	C	4
96	C	C	5
97	D	D	5
98	C	E	3
99	D	D	5
100	D	C	5
101	E	E	5
102	E	C	5
103	C	C	3
104	B	B	4
105	A	D	5
106	E	E	3
107	B	B	4
108	E	E	5
109	A	A	5
110	C	C	5
111	B	B	5
112	A	A	5
113	E	E	5
114	D	E	5
115	C	C	5
116	A	A	5
117	B	C	5
118	C	E	5
119	C	C	5
120	E	E	5

**Table 2:** Effectiveness of ChatGPT-4o depending on the question type: clinical cases vs. others Data are presented as absolute numbers (N) and percentages (%). No statistically significant differences were found between the groups (p = 0.709; χ² = 0.139).

Question type	Correct answer N (%)	Incorrect answer N (%)	p-value	χ² value
Clinical cases	40 (80)	10 (20)	0.709	0.139
Other	54 (77.1)	16 (22.9)		

**Figure 2:**
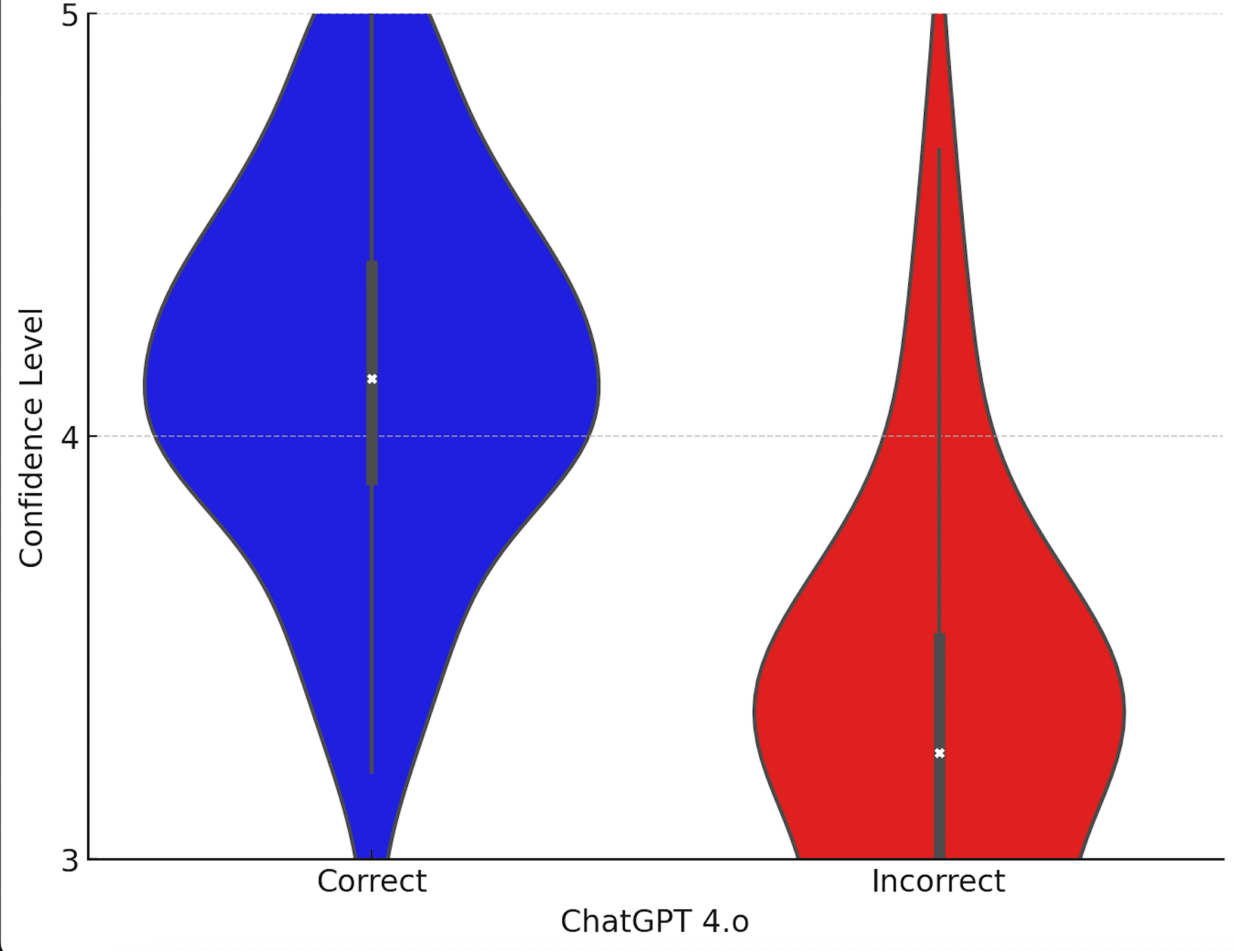
The declared confidence level of the ChatGPT-4o model depending on the accuracy of the answers The values are presented separately for correct and incorrect responses. A higher confidence level was observed for correct answers.

Based on the analysis of the declared confidence levels in the answers provided by the ChatGPT-4o model, a significant difference was observed between correct and incorrect answers. The graph shows the distribution of confidence levels (on a scale of 1 to 5) for both categories of answers. The model exhibited a significantly higher confidence level for correct answers, while lower confidence values dominated in incorrect answers (Figure 2).

## Discussion

The PES exam in ophthalmology is one of the key stages in the process of acquiring specialization for doctors in this field. It is characterized by a high level of difficulty and requires both theoretical knowledge and practical problem-solving skills in clinical scenarios. This study analyzes how the advanced language model ChatGPT-4o performed on ophthalmology exam tasks. The model achieved a score of 78.3%, which exceeds the passing threshold and is significantly higher than the results obtained by earlier versions of ChatGPT models in similar studies in other medical fields. This study is the only one available in the literature analyzing the effectiveness of ChatGPT for the PES exam in ophthalmology. Our results align with the observations published by Jaworski et al. in the context of the Final Medical Examination (LEK) exam, where ChatGPT-4 achieved 77.6%, surpassing the passing threshold (56%) and consistently answering clinical and general questions [6]. A similar effectiveness (78.3%) was observed in the PES exam for ophthalmology. It is worth noting that newer versions of the model - from ChatGPT-3.5 to 4 and 4o - show a clear increase in effectiveness, indicating progress in the medical understanding of AI models. However, unlike the results from the LEK, in our study, the model’s confidence level showed a significant correlation with the accuracy of its answers. This distribution suggests that the model is partially able to recognize its competencies and signal uncertainty, which could have practical implications when used as a tool for supporting learning or clinical decision-making. It is important to emphasize that these differences were statistically significant, as confirmed by the Mann-Whitney U test. The consistency of these results across different exams underscores the potential of AI as a tool to support medical education, but also indicates the need for cautious implementation of AI systems with appropriate expert oversight. Our study results can also be compared with observations published in a paper on the cardiology specialization exam [7]. This study evaluated the effectiveness of ChatGPT-3.5 and ChatGPT-4.0 in solving 120 questions from the PES exam in cardiology. ChatGPT-4.0 achieved an effectiveness rate of 76.7%, which is comparable to the result obtained in our ophthalmology study (78.3%). In both cases, the model exceeded the passing threshold of 60%, confirming its educational potential even in more challenging specialist exams. A key common point in both studies is the high accuracy of answers to questions based on clinical knowledge. In the cardiology paper, the authors observed that the GPT-4 model performed better with case-based questions than with questions requiring the memorization of detailed numerical data or classifications. Blecha et al. conducted a study aimed at evaluating the performance of the DeepSeek-R1 (DeepSeek AI, Zhejiang, China) and ChatGPT-4o models on the Infectious Diseases PES exam. They demonstrated that both models are capable of passing the exam, making them useful educational tools [8]. The capabilities of different ChatGPT versions in taking medical exams have also been examined, referring among others to allergology [9], dermatology [10], and nuclear medicine [11]. A similar pattern is observed in our analysis - clinical questions did not significantly differ in effectiveness from other types, and the model’s confidence level was significantly higher with correct answers. Ultimately, it should be noted that while the results are promising, there are still limitations associated with the use of language models in medical education. These models can generate seemingly convincing but incorrect answers, which, in a clinical context, could lead to undesirable decisions. Therefore, their role should be considered as supportive - in education, training, and case analysis - with maintaining appropriate academic supervision.

## Conclusions

The ChatGPT-4o model demonstrated high effectiveness in solving PES ophthalmology exam questions in the Spring 2025 session, achieving a result significantly exceeding the passing threshold. The results suggest that such AI models can be valuable tools in supporting specialist education, especially in terms of self-repetition, exam simulations, and testing knowledge levels in real-world conditions. Due to their ability to generate human-like responses and self-assess confidence, models like ChatGPT-4o can also function as interactive teaching assistants, aiding the learning process by identifying knowledge gaps and providing targeted feedback. However, their integration into the medical education system requires further research - both in terms of validating effectiveness in other medical fields and developing safe and ethical implementation strategies. It is also necessary to develop standards for expert oversight of content generated by AI models and define the limits of their application, ensuring they support the educational process without replacing critical thinking and expert supervision.
